# Magnetic resonance direct thrombus imaging to distinguish acute pulmonary embolism from chronic thromboembolic pulmonary hypertension: a case report

**DOI:** 10.1093/ehjcr/ytaf555

**Published:** 2025-11-01

**Authors:** Akihiro Okamoto, Tomohiro Yamaguchi, Takanori Yamazaki, Shoichi Ehara, Daiju Fukuda

**Affiliations:** Department of Cardiovascular Medicine, Osaka Metropolitan University Graduate School of Medicine, 1-4-3 Asahimachi, Abeno-ku, Osaka 545-8585, Japan; Department of Cardiovascular Medicine, Osaka Metropolitan University Graduate School of Medicine, 1-4-3 Asahimachi, Abeno-ku, Osaka 545-8585, Japan; Department of Cardiovascular Medicine, Osaka Metropolitan University Graduate School of Medicine, 1-4-3 Asahimachi, Abeno-ku, Osaka 545-8585, Japan; Department of Intensive Care Medicine, Osaka Metropolitan University Graduate School of Medicine, 1-4-3 Asahimachi, Abeno-ku, Osaka 545-8585, Japan; Department of Cardiovascular Medicine, Osaka Metropolitan University Graduate School of Medicine, 1-4-3 Asahimachi, Abeno-ku, Osaka 545-8585, Japan

**Keywords:** Acute pulmonary embolism, Chronic thromboembolic pulmonary hypertension, Magnetic resonance direct thrombus imaging, Case report

## Abstract

**Background:**

Acute pulmonary embolism and chronic thromboembolic pulmonary hypertension are both pulmonary thrombotic diseases. However, it is difficult to differentiate between them in some cases, complicating the choice of suitable treatment. Computed tomography pulmonary angiography and D-dimer measurement are helpful for diagnosing these conditions; however, these examinations cannot estimate the efficacy of anticoagulants in the pulmonary artery.

**Case summary:**

The present case was an 83-year-old woman who had experienced dyspnoea on exertion for 2 months. Computed tomography pulmonary angiography revealed large mural thrombus in the proximal right pulmonary artery, and serum D-dimer concentration was slightly elevated (2.6 µg/mL). Magnetic resonance direct thrombus imaging showed high-intensity signals on T1-weighted images in the area corresponding to the thrombi detected by computed tomography pulmonary angiography. We predicted that the proximal pulmonary artery thrombi were not chronic, and we prescribed an anticoagulant for initial treatment. Seven months later, computed tomography pulmonary angiography showed disappearance of the mural thrombus, and there were no high-intensity signals in the pulmonary artery on magnetic resonance direct thrombus imaging. We diagnosed chronic thromboembolic pulmonary hypertension without proximal lesions after 6 months of anticoagulation therapy according to the elevated mean pulmonary arterial pressure of 32 mmHg. We performed three sessions of balloon pulmonary angioplasty to treat the distal lesions. The patient is well, and her haemodynamics have significantly improved.

**Discussion:**

Magnetic resonance direct thrombus imaging has the potential to differentiate between acute pulmonary embolism and chronic thromboembolic pulmonary hypertension. Further studies are required because this is only a case report.

Learning points

- Acute pulmonary embolism (PE) and chronic thromboembolic pulmonary hypertension (CTEPH) are closely related diseases, and evaluating thrombus age is very important in determining suitable treatment.
- Magnetic resonance direct thrombus imaging may help identify thrombus age in ambiguous PE vs. CTEPH cases, potentially informing choice between anticoagulation and pulmonary hypertension-targeted therapy.

## Introduction

Acute pulmonary embolism (PE) is a potentially fatal cardiovascular disease characterized by thrombi development, sometimes resulting in early in-hospital death.^1,2^ Chronic thromboembolic pulmonary hypertension (CTEPH) is a fatal late sequela of acute PE, whereas pre-existing CTEPH is missed in some patients suspected of acute PE.^1,2^ In cases of acute PE combined with chronic thrombi, the accurate estimation for the response to anticoagulant therapies is an important but challenging issue when considering appropriate treatments. Computed tomography pulmonary angiography (CTPA) and D-dimer concentration can provide helpful information to diagnose these conditions; however, they are not always effective in estimating the response to anticoagulant therapies.^1^

## Summary figure

**Figure:**
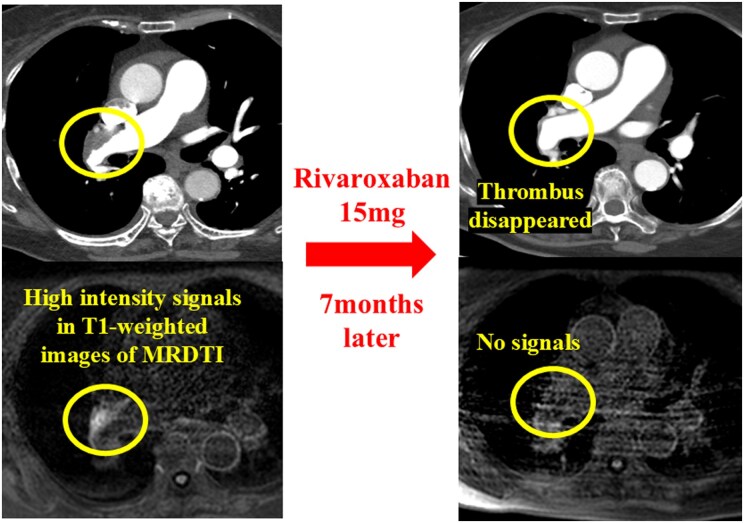


## Case presentation

**Figure 1:**
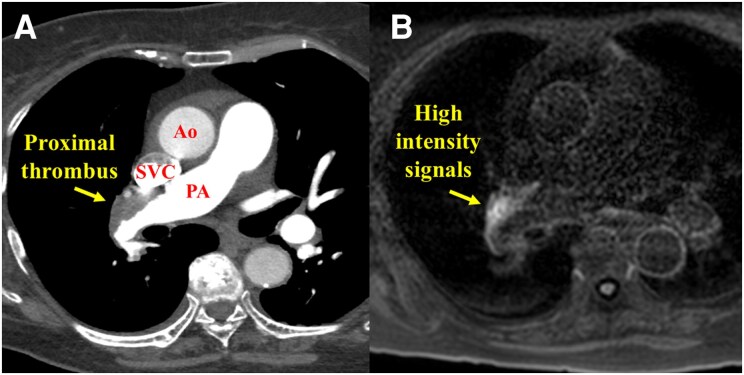
CTPA and MRDTI images at the patient’s initial referral. (*A*) Large mural thrombi were seen in the proximal right pulmonary artery on CTPA. (*B*) High-intensity signal on MRDTI at the area of the mural thrombi detected by CTPA, which suggested a possibility that this thrombus was not chronic. CTPA, computed tomography pulmonary angiography; MRDTI, magnetic resonance direct thrombus imaging; Ao, ascending aorta; SVC, superior vena cava; PA; pulmonary artery.

**Figure 2:**
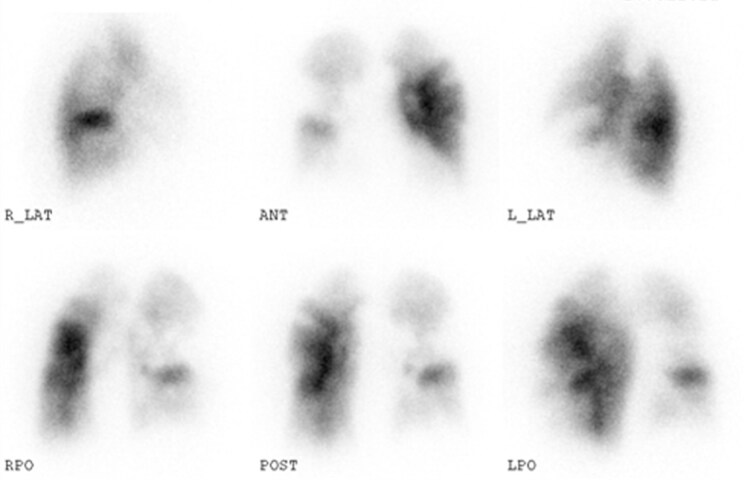
Lung perfusion scintigraphy images. There were some segmental defects in both lungs.

**Figure 3:**
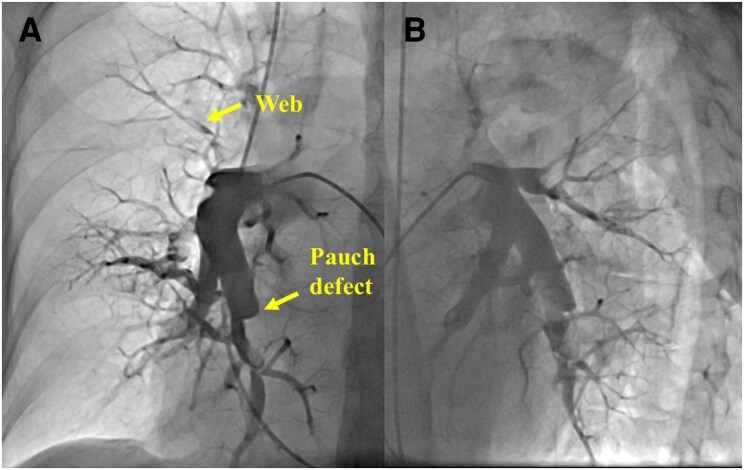
Pulmonary angiography images. (*A*) Anterior–posterior view. (*B*) Left anterior oblique view (60°). Pulmonary angiography suggested that her diagnosis was acute PE on background of undiagnosed chronic thromboembolic pulmonary hypertension, illustrating webs and pouch defects.

The patient was an 83-year-old woman who had pitting oedema in both legs and gradually worsened dyspnoea on exertion without sudden onset from 2 months ago and was referred to our hospital. She had been treated for diabetes using insulin. There were no other risk factors for PE. The patient’s blood pressure was 116/78 mmHg, and her heart rate was 88 b.p.m. with sinus rhythm. Her respiratory rate was 16 breaths/min, and her oxygen saturation decreased to 92% on room air. In her physical examination, there were no other significant findings in the lungs and abdomen. Blood tests showed remarkably elevated brain natriuretic peptide (378.3 pg/mL—reference normal values < 18.4 pg/mL) and slightly elevated D-dimer (2.6 µg/mL—reference normal values < 1.0 µg/mL). Transthoracic echocardiography showed right ventricular dilatation and moderate tricuspid regurgitation with a high pressure gradient of 55 mmHg. Computed tomography pulmonary angiography revealed large mural thrombus in the proximal right pulmonary artery, morphologically suggestive of chronic thrombus (*Figure 1A*). Lung perfusion scintigraphy showed perfusion defects in both lung fields (*Figure 2*). Right heart catheterization indicated high mean pulmonary artery pressure (mPAP; 41 mmHg) and high pulmonary vascular resistance (PVR; 15.0 Wood units), as well as a low cardiac index (1.22 L/min/m²). Pulmonary angiography suggested CTEPH, illustrating webs and pouch defects (*Figure 3*).^1,2^ The additional examination was needed because these popular testing could not provide a definite diagnosis of her, i.e. acute PE and CTEPH.

Magnetic resonance direct thrombus imaging (MRDTI) indicated high-intensity signals in the areas corresponding to the PA thrombi detected by CTPA (*Figure 1B*). Magnetic resonance direct thrombus imaging was obtained using a 1.5 T magnetic resonance system (Achieva, Philips Medical Systems, Best, the Netherlands). The patient was breathing freely, as shown by the three-dimensional T1-weighted imaging inversion-recovery gradient-echo sequence. A black blood condition was identified by the Look-Locker sequence, fat-suppressed sequence, and radial k-space sampling in the Y–Z plane. Magnetic resonance direct thrombus imaging suggested that the thrombi in the right proximal PA were not chronic. Thus, we diagnosed acute PE on background of undiagnosed distal-type CTEPH. We prescribed rivaroxaban 15 mg twice daily for 3 weeks, followed by 15 mg once daily, as the initial treatment.

**Figure 4:**
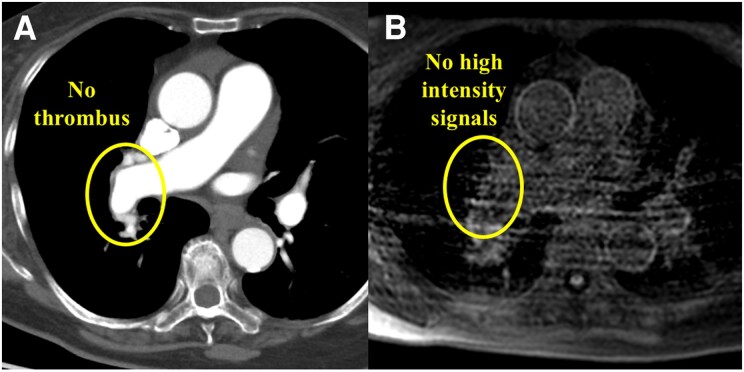
CTPA and MRDTI images after 6 months of anticoagulation therapy. (*A*) No thrombi could be seen in the proximal PA on CTPA. (*B*) The MRDTI image shows no high-intensity signal in the area corresponding to the thrombi detected by CTPA at the patient’s initial referral. CTPA, computed tomography pulmonary angiography; MRDTI, magnetic resonance direct thrombus imaging; PA, pulmonary artery.

Three months after the initiation of anticoagulation therapy, right heart catheterization showed residual PH (mPAP, 45 mmHg; PVR, 11.9 Wood units; cardiac index, 1.44 L/min/m^2^). We prescribed riociguat 6.0 mg due to her severe symptoms and sustained low cardiac output. Seven months after the patient’s initial referral to our hospital, CTPA indicated disappearance of the mural thrombi in the proximal PA, which initially showed high-intensity signals on MRDTI. This time, there were no residual high-intensity signals in the PA on MRDTI (*Figure 4*). We finally diagnosed distal-type CTEPH, considering the elevated mPAP of 32 mmHg, after 7 months of anticoagulation therapy. Consequently, we performed three sessions of balloon pulmonary angioplasty (BPA) to treat the distal lesions.

The patient is well without home oxygen therapy, and her pulmonary haemodynamics have improved (mPAP, 16 mmHg; cardiac index, 1.6 L/min/m^2^; PVR, 4.43 Wood units). Her 6-min walking distance was improved from 196 to 345 m.

## Discussion

Estimating the response to anticoagulant therapies is one of the challenging issues when managing patients with pulmonary thrombosis.^1,2^ We previously reported a possible difference in signal intensity on MRDTI between patients with acute PE and those with CTEPH.^3^ However, its clinical importance was not considered in detail. This is the first report to demonstrate the possible efficacy of MRDTI in predicting the dissolvement of thrombi in a patient with acute PE on background of undiagnosed CTEPH.

Chronic thromboembolic pulmonary hypertension is a sequela of acute PE; however, it is sometimes difficult to differentiate between CTEPH and PE clinically in patients without acute symptoms, which makes the choice of appropriate treatment more difficult.^4^ Pulmonary hypertension-targeted therapy, pulmonary endarterectomy, and BPA are recommended for CTEPH.^2^ However, these treatments are not recommended for patients with acute PE. Pulmonary endarterectomy is recommended for patients with CTEPH with proximal lesions.^2^

Although ring-like stenoses, webs/slits, and chronic total occlusion on CTPA images can help to reach the diagnosis of CTEPH, these morphological features sometimes cannot provide an accurate diagnosis because some patients with acute PE have pre-existing CTEPH.^1,5,6^ Serum D-dimer measurement is useful to exclude acute PE; however, it cannot clinically differentiate between acute PE and CTEPH.^1^ In the present case, thrombus morphology of CTPA and slightly elevated D-dimer level suggested that her diagnosis was not a simple acute PE.^1,7,8^ Pulmonary angiography showed some CTEPH-specific findings, such as webs and pouch defects (*Figure 3*). Based on these findings, she was initially diagnosed as acute PE on background of undiagnosed CTEPH. In considering the preferable treatments for her, the estimation of the response to anticoagulants was important to determine the indication of pulmonary endarterectomy. And its earlier differentiation was demanded because of her unstable and decompensated haemodynamics. Magnetic resonance direct thrombus imaging could help the diagnosis and following treatments.

The advent of carotid and coronary plaque characterization with non-contrast T1-weighted magnetic resonance imaging has facilitated plaque imaging based on the presence of high-intensity signals within the thrombus or intraplaque haemorrhage caused by methaemoglobin T1 shortening.^9^ Ehara *et al*. showed that high-intensity signals were more frequently observed in obstructed coronary lesions with shorter occlusion durations, and the signal intensity of the target area and the cardiac muscle ratio were higher for newer occlusive lesions.^10^

Some patients clinically diagnosed as acute PE had the combination of acute and chronic thrombi, which is characterized as web and bands.^11^ Thus, there is a possibility that the meaning of high signals in MRDTI is different from arterial thrombus. In the context of venous thrombosis, Tan *et al*.^12^ reported the usefulness of MRDTI for the accurate differentiation of acute and chronic thrombi in lower-limb deep veins compared with compression ultrasonography. And MRDTI has been reported to be a useful and effective technique for the diagnosis of deep vein thrombosis.^12–14^ These insights suggest a possibility that MRDTI could predict the response to anticoagulants in pulmonary thrombi. This case highlights the potential utility for estimating the response to anticoagulants in PE; however, further validation cohorts are essential.

Magnetic resonance direct thrombus imaging may provide us the information regarding the response to anticoagulants in patients with acute PE. However, this imaging technique has some clinical limitations. First, the image quality of MRDTI depends on the patient’s respiratory condition. Second, patients with fatal condition are not suitable for this technique. Finally, further robust validations in prospective studies are required before integrating MRDTI into routine practice because this is only a case report.

## Lead author biography


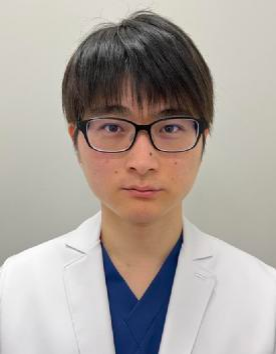


Dr Akihiro Okamoto is a senior resident at Osaka Metropolitan University Graduate School of Medicine.

## Data Availability

Data sharing is not applicable to this article because no datasets were generated or analysed during this report.

## Notes

**Consent:** The authors confirm that written consent for submission and publication of this case report including images and associated text has been obtained from the patient in line with the COPE guidelines.

**Funding:** None declared.
